# CUL4B Promotes Temozolomide Resistance in Gliomas by Epigenetically Repressing *CDNK1A* Transcription

**DOI:** 10.3389/fonc.2021.638802

**Published:** 2021-04-02

**Authors:** Xiang Ye, Xiaochen Liu, Min Gao, Li Gong, Fei Tian, Yangli Shen, Huili Hu, Gongping Sun, Yongxin Zou, Yaoqin Gong

**Affiliations:** ^1^ Key Laboratory of Experimental Teratology of Ministry of Education, Department of Medical Genetics, School of Basic Medical Sciences, Cheeloo College of Medicine, Shandong University, Jinan, China; ^2^ Department of Geriatric Medicine, Qilu Hospital of Shandong University, Jinan, China; ^3^ Department of Obstetrics and Gynecology, Qilu Hospital of Shandong University, Jinan, China; ^4^ Key Laboratory for Experimental Teratology of Ministry of Education, Department of Histoembryology, School of Basic Medical Sciences, Cheeloo College of Medicine, Shandong University, Jinan, China

**Keywords:** CUL4B, p21, temozolomide resistance, glioma, epigenetic regulation

## Abstract

Resistance to temozolomide (TMZ), the first-line chemotherapeutic drug for glioblastoma (GBM) and anaplastic gliomas, is one of the most significant obstacles in clinical treatment. TMZ resistance is regulated by complex genetic and epigenetic networks. Understanding the mechanisms of TMZ resistance can help to identify novel drug targets and more effective therapies. CUL4B has been shown to be upregulated and promotes progression and chemoresistance in several cancer types. However, its regulatory effect and mechanisms on TMZ resistance have not been elucidated. The aim of this study was to decipher the role and mechanism of CUL4B in TMZ resistance. Western blot and public datasets analysis showed that CUL4B was upregulated in glioma specimens. CUL4B elevation positively correlated with advanced pathological stage, tumor recurrence, malignant molecular subtype and poor survival in glioma patients receiving TMZ treatment. CUL4B expression was correlated with TMZ resistance in GBM cell lines. Knocking down CUL4B restored TMZ sensitivity, while upregulation of CUL4B promoted TMZ resistance in GBM cells. By employing senescence β-galactosidase staining, quantitative reverse transcription PCR and Chromatin immunoprecipitation experiments, we found that CUL4B coordinated histone deacetylase (HDAC) to co-occupy the *CDKN1A* promoter and epigenetically silenced *CDKN1A* transcription, leading to attenuation of TMZ-induced senescence and rendering the GBM cells TMZ resistance. Collectively, our findings identify a novel mechanism by which GBM cells develop resistance to TMZ and suggest that CUL4B inhibition may be beneficial for overcoming resistance.

## Introduction

Gliomas are the most common malignant brain tumors worldwide, with overall morbidity ranging from 4.67 to 5.73 per 100 000 persons (1, 2). It is categorized in four grades, I-IV, based on histological criteria, phenotype and genotype. Glioblastoma (GBM), grade IV glioma, is the most aggressive and lethal sub-type of glioma, accounting for the majority (56.1%) (1). Despite extensive treatment including surgery, radiotherapy and chemotherapy, the prognosis of GBM is still poor, with a survival rate of only 5.8% at 5 years postdiagnosis (3, 4). Temozolomide (TMZ), a DNA-alkylating agent that causes persistent DNA strand breaks and replication fork collapse (5–7), is currently used as the first-line chemotherapeutic drug for GBMs and anaplastic gliomas (8, 9). Owing to the introduction of TMZ and adoption of radiotherapy followed by adjuvant TMZ, the median survival of patients with GBM has been prolonged from 12.1 to 14.6 months (10). However, the overall clinical effect of this regimen is still disappointing. Tumor progression occurs in over 40% of patients who receive TMZ therapy (11–13). Failure to generate more effective treatment by TMZ is largely due to intrinsic or acquired resistance to TMZ. Therefore, there is an urgent need to address the mechanism underlying TMZ resistance and to identify potential predictive biomarkers and novel therapeutic targets in gliomas.

Although considerable attention has been focused on several key molecular events, such as expression of O6-methylguanine-DNA methyltransferase (MGMT), mutations in IDH, ATRX and EGFR gene, other mechanisms, such as cancer stem-like cells, Poly(ADP)-ribose polymerase, are also associated with TMZ resistance in GBMs (14–17), suggesting the complexity of the mechanism underlying TMZ resistance. The therapeutic action of TMZ to GBM is exerted by triggering DNA damage, which in turn induces apoptosis and senescence (18). Indeed, TMZ induces senescence at high level in glioma cells (7). Senescence is considered to be a clinically favorable response to chemotherapy. Senescence permanently blocks the proliferation of tumor cells, and the senescent tumor cells are rapidly cleared by immune cells, resulting in efficient tumor regression. Growing evidence shows that therapy-induced senescence in tumors is associated with improved clinical outcomes (19–21), and inducing senescence in cancers has recently been explored as a therapeutic strategy with the potential to reduce cytotoxicity (22, 23), and potentiate drug combinations (24). Therefore, elucidation of the mechanisms underlying TMZ-induced senescence could provide attractive novel molecular targets for glioma therapy.

CUL4B is a scaffold protein of Cullin 4B-Ring E3 ubiquitin ligases (CRL4B), which are involved in a wide variety of physiologically and developmentally processes (25–27). Our previous studies show that CUL4B could exert its functions partly by catalyzing H2AK119 monoubiquitination, leading to the transcriptional repression of targeted genes (27–30). The role of CUL4B in cancers is diverse and context-dependent. While CUL4B acts as an oncogene in many solid tumors (31–36), in hematopoietic cells CUL4B exerts a tumor-suppressive effect by restricting the accumulation and function of myeloid-derived suppressor cells (37, 38).

Previous studies have demonstrated that CUL4B is related to chemoresistance in lymphoblastoid cells, non-small-cell lung cancer cells, osteosarcoma and bladder cancer cells (29, 39, 40). It has been reported that CUL4B promotes proliferation of glioma cells (41). However, whether CUL4B is involved in TMZ resistance in glioma is unknown. In this study, we aim to investigate the role of CUL4B in TMZ resistance. Induction of senescence is an important mechanism by which TMZ treats tumors. Previously, we found that CUL4B could impede stress-induced cellular senescence (42). Therefore, we hypothesize that CUL4B may promote TMZ resistance through enhancing TMZ-induced senescence in glioma cells. To verify this hypothesis, we used glioma patient samples to analyze the association between CUL4B expression and TMZ resistance, and GBM cell lines to further investigate the role of CUL4B in TMZ resistance and the underlying mechanism.

## Materials and Methods

### Tissue Specimens

Ethical approval was obtained from School of Basic Medical Sciences, Cheeloo College of Medicine, Shandong University. Fresh glioma tissue and corresponding peritumor tissue specimens were obtained from 29 glioma patients at Qilu Hospital of Shandong University from February 2016 to June 2019. Each case was collected at the time of surgery after informed consent. All tissue samples were examined and classified under the management of experienced pathologists. Patients were enrolled in the study if their diagnosis was confirmed histologically by two neuropathologists based on the 2007 WHO classification guidelines. The data of the patients are presented in **Supplementary Table S1**.

### Western Blot

Preparation of protein extracts and Western blot analysis was performed as described previously (25, 26). In brief, the protein extracts of cells or tissue samples were lysed using cell lysis buffer (Beyotime Biotechnology, Shanghai, China). After centrifugation, supernatants were collected. The extracts were quantified using a BCA protein assay kit (Beyotime Biotechnology, Shanghai, China). For Western blot, equal amounts of extracts were loaded on 10% SDS-polyacrylamide gels, electrophoresed, and blotted onto PVDF membranes (GE Healthcare). The membrane was blocked with 5% skimmed milk, followed by incubation with specific primary antibodies overnight at 4°C. Then the membranes were incubated with HRP-conjugated secondary antibodies and detected using the ECL PLUS kit (GE Healthcare). The protein bands were quantified using NIH image analysis software (ImageJ Version 2.0, National Institutes of Health, Bethesda, MD, USA). The antibodies used are listed in **Supplementary Table S2**.

### Bioinformatic Analysis Using Online Databases

Gene expression data and clinical characteristics (tumor grade, age at diagnosis, survival time, censored status, and treatment history) were acquired from the Chinese Glioma Genome Atlas (CGGA) database (http://www.cgga.org.cn). Data from mRNAseq_325 (containing 325 samples) was used for the gene expression analysis of low-grade gliomas (LGGs, grade II) and high-grade gliomas (HGGs, grade III and IV GBM). mRNA-array_301 data (containing 301 samples) was employed for GBM subtypes. Other analyses were implemented using mRNAseq_693 data (containing 693 samples). Disease-specific overall survival (OS) was calculated from the date of diagnosis until disease-caused death or end of follow-up. Differences in expression features between different groups were determined using the Student’s t-tests. Kaplan−Meier curves were developed *via* GraphPad Prism 8 (GraphPad Software Inc., San Diego, CA, USA), comparing overall survival rates between patients with and without the genetic alterations of interest. The log rank tests were conducted to assess the prognostic significance. The detail information was supplemented in **Supplementary Table S3**.

Clinical information for GBM patients (n = 41) and corresponding tumors (n = 42) was downloaded from the Ivy Glioblastoma Atlas Project (Ivy GAP) (43). The Ivy GAP RNAseq dataset was used to analyze CUL4B expression in different GBM regions. Detailed information of datasets was shown in **Supplementary Table S4**.

mRNA expression profiles in GSE72951 (44) from the publicly available the Gene Expression Omnibus (GEO) database (https://www.ncbi.nlm.nih.gov/geo), which had been collected using the Illumina GPL14951 platform (HumanHT-12 WG-DASL V4.0 R2 expression beadchip) were analyzed. GSE72951 includes 112 GBM surgical specimens. mRNA expression profiles which had been collected using the GPL8300 platform (Affymetrix Human Genome U95 Version 2 Array) in GSE13041 (45), were analyzed. The GSE13041 dataset includes 267 primary GBM surgical specimens. Detailed information of datasets was shown in **Supplementary Table S5**.

### Cell Culture and Manipulation

The human GBM cell lines U87, A172, U118, T98, and HEK293T (CRL-3216) cell lines were obtained from the American Type Culture Collection. U251 and SHG44 were obtained from Cell Bank, Chinese Academy of Sciences, Shanghai, China. U87, A172, U118, T98, HEK293T, and U251 cells were grown in DMEM (Gibco, Grand Island, NY, USA). SHG44 cells were cultured in RPMI-1640 medium (Gibco, Grand Island, NY, USA). All the media were supplemented with 10% fetal bovine serum (FBS), and all cell cultures were maintained at 37°C in a 5% CO_2_ incubator. CUL4B stable knockdown, stable overexpressed, and control cells were generated as previously described (29, 42). p21 overexpression plasmid was purchased from Shanghai Jikai Gene Co., Ltd. Transfection of plasmids was performed according to standard protocols using Lipofectamine 2000 (Invitrogen, Carlsbad, CA, USA).

### Quantitative Reverse Transcription-Polymerase Chain Reaction (qRT-PCR)

Extraction of total RNA and qRT-PCR used to detect CUL4B and p21 mRNA levels was performed as previously described (25, 30). In brief, total RNA from cultured cells was isolated using TRIzol reagent (Invitrogen, Carlsbad, CA, USA) according to the manufacturer’s instructions. Total RNA was transcribed to generate cDNA using PrimeScript RT Reagent Kit (Takara Co, Otsu, Japan) with random hexamers as primers. Real-time quantitative PCR (qPCR) was performed using the LightCycler 480 system (Roche, Mannheim, Germany). The mRNA levels were measured by a SYBR Green I assay using SYBR Green Universal PCR Master Mix (Applied Biosystems) according to the manufacturer’s instructions. Each reaction was run in triplicate and in parallel. Primer sequences designed for qPCR are listed in **Supplementary Table S6**.

### Cell Viability Determination and Colony Formation Assays

Cell viability was determined by CCK-8. Colony formation assays were employed to measure the sensitivity of TMZ. The CCK-8 and colony formation assays were performed as previously described (29). For CCK-8 assays, 1 × 10^3^ cells per well were plated into a 96-well plate. After 24 h, the cells were treated with fresh medium including various concentrations of TMZ (SelleckChem, Houston TX, USA) for 72 h. Then, CCK-8 (Beyotime Biotechnology, Shanghai, China) was added to each well for 40 min before determined the absorption values at 450 nm. The IC50 was calculated using GraphPad Prism 8 software. Colony forming ability was assessed using a colony formation assay. Cells were seeded into 6cm dishes and then incubated with TMZ for two weeks. After the medium was removed, cells were fixed with methanol, and then stained using crystal-violet staining solution, finally photographed. The number of colonies was counted.

### Animal Tumor Models

Tumor xenografts were used to determine the role of CUL4B in TMZ resistance *in vivo*. Female nude mice (BALB/c-nu; 5 weeks old) were purchased from Vital River Laboratory Animal Technology Co, Ltd and maintained in a specific pathogen-free environment. The animal protocol was approved by the Shandong University Animal Care Committee and all care procedures were in compliance with institutional guidelines. 5×10^6^ CUL4B knockdown or control U118 cells were administrated into the subcutaneous tissues of both flanks of the nude mice. Tumor size was measured every 6 days after tumor inoculation. When the tumors reached to a noticeable size(150-200mm^3^), the tumor-bearing mice were treated with TMZ (50 mg/kg/day) intraperitoneally for 5 days per week for two cycles. Mice were sacrificed 9 weeks after inoculation, and tumors were excised.

### Immunohistochemistry (IHC)

In brief, xenograft tumor tissues were dissected from nude mice and fixed in 4% PFA at 4°C overnight, then dehydrated, embedded in paraffin, and sectioned. After performing deparaffinization and rehydration, the sections were boiled in sodium citrate buffer for 10 min for antigen recovery, and immersed in 3% H_2_O_2_ for 10 min to quench endogenous peroxidase. After blocked with 10% serum at 37°C for 1 h to reduce nonspecific staining, sections were then incubated overnight at 4°C with primary antibodies (The antibodies used are listed in **Supplementary Table S2**). HRP-conjugated secondary antibodies were applied on the second day and visualized using DAB. Sections were counterstained with hematoxylin and mounted on glass slides.

### SA-β-Galactosidase Staining

To measure cellular senescence, the SA-β-galactosidase Staining (SA-β-gal) activity was assessed by the SA-β-gal staining kit (Beyotime Biotechnology, Shanghai, China) following the manufacturer’s instructions. Briefly, Cells were prepared with the indicated treatments. Cells grown in 6-well plates were fixed with Fixative Solution for 15 minutes at room temperature, and then incubated with β-Galactosidase Staining Solution at 37 °C in a dry incubator without CO_2_ overnight. The percentage of SA-β-Gal positive cells was represented by the ratio between the number of blue-colored cells and the number of total cells. A minimum of 500 cells were counted for each sample.

### Chromatin Immunoprecipitation Assays

To detect the binding of CUL4B and HDACs to p21 promoter, Chromatin immunoprecipitations (ChIPs) were performed as the previous description (27, 46). Briefly, the U87 cells were crosslinked with formaldehyde, sonicated, pre-cleared and immunoprecipitated with specific antibodies at 4°C overnight. Subsequently, Protein-A/G–Sepharose CL-4B beads were prepared and added to bind with the antibody for 2 h at 4°C. Then, complexes were washed, and the DNA was extracted and precipitated. Using PCR primers specific for *CDNK1A* promoter, the enrichment of the DNA template was analyzed. The primers and antibodies are listed in **Supplementary Table S7**.

### Statistical Analysis

Statistical analysis was performed using Statistical Package for Social Science (SPSS) version 20.0 and GraphPad Prism 8 software. Each experiment was performed at least in triplicate with three independent sets of culture. All results were expressed as mean ± standard deviation (SD). The Student’s t-test and One-way ANOVA were used for comparison between and among groups, respectively. Survival curves were plotted by Kaplan−Meier analysis and log-rank test was used for analysis. p<0.05 was considered statistically significant.

## Results

### CUL4B Expression Is Upregulated and Associated with Poor Prognosis and TMZ Resistance in Glioma

**Figure 1:**
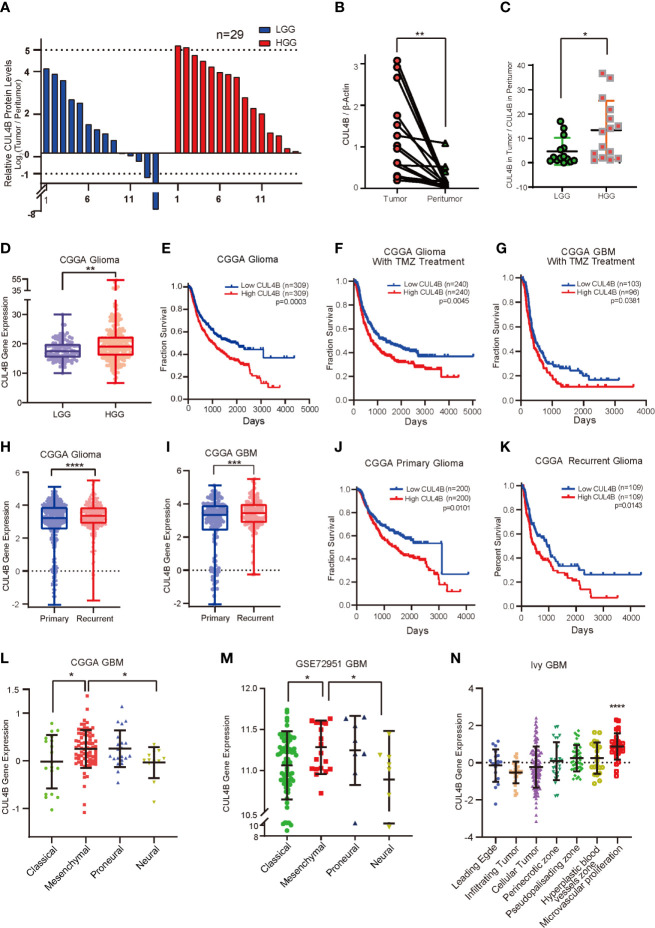
CUL4B expression is upregulated and associated with poor prognosis and TMZ resistance in glioma. **(A)** CUL4B expression levels in gliomas relative to those in paired non-malignant tissues, as determined by Western blot. **(B)** CUL4B levels in the HGGs and paired adjacent non-malignant tissues. The value represents the densitometry value of CUL4B band on immunoblots normalized with that of β-actin. **(C)** CUL4B expression in LGG and HGG patients. The value represents the densitometry value of CUL4B band in tumor tissue compare to that in paratumor on immunoblots normalized to that of β-actin. **(D)** Expression of CUL4B mRNA in LGG and HGG obtained from the CGGA database. **(E–G)** Kaplan–Meier analysis with a log-rank test for the overall survival of all glioma patients **(E)**, glioma patients with TMZ therapy **(F)** and GBM patients with TMZ therapy **(G)** from the CGGA database. **(H)** Expression of CUL4B in primary and recurrent gliomas obtained from the CGGA database. **(I)** CUL4B mRNA levels in primary and recurrent GBMs obtained from the CGGA database. **(J, K)** Kaplan-Meier curves for overall survival of primary glioma patients **(J)** and recurrent glioma patients **(K)** from the CGGA database. **(L, M)** CUL4B mRNA expression in four subtypes of GBM obtained from CGGA **(L)** and GSE27951 **(M)** dataset. **(N)** CUL4B expressions in tumor samples from the Ivy Glioblastoma Atlas. Each point corresponds to an individual laser-micro dissected sample. (LGG, low-grade glioma; HGG, high-grade glioma; GBM, glioblastoma; CGGA, Chinese Glioma Genome Atlas. ****P < 0.0001, ***P < 0.001, **P < 0.01, *P < 0.05).

To investigate if CUL4B is associated with the progression of glioma, we first examined CUL4B protein levels in 29 tumor samples and paired adjacent non-malignant tissues by Western blot (**Supplementary Figure 1A**). CUL4B level in low-grade gliomas (LGGs, grade II) was slightly higher than that of adjacent non-malignant tissues (**Figure 1A** and **Supplementary Figure 1B**), whereas CUL4B expression was notably elevated in more aggressive high-grade gliomas (HGGs, grade III and IV GBM), comparing to the paired non-cancerous tissues (**Figures 1A, B**). Moreover, HGG tumors exhibited higher expression of CUL4B compared with LGG tumors (**Figure 1C**). The association between CUL4B and glioma were further analyzed using CGGA database. The results confirmed that CUL4B expression was significantly higher in HGG than that in LGG samples (**Figure 1D**). These data suggest that CUL4B is upregulated in glioma tissues and positively correlated with tumor grade.

To gain more insight into the clinical relevance of CUL4B expression, we investigated the relationship between CUL4B expression level and prognosis using CGGA and GSE13041 database. Survival analysis revealed that glioma patients with higher CUL4B expression levels exhibited shorter survival durations (**Figure 1E**). Similar results were observed in the HGG (**Supplementary Figure 2A**) and GBM patients (**Supplementary Figure 2B**). Given the importance of CUL4B expression in patient survival and the critical roles of TMZ resistance in glioma prognosis, we speculated that CUL4B might be involved in TMZ resistance. Analysis of CUL4B expression using CCGA database revealed that high CUL4B expression correlated with poor survival in glioma patients receiving TMZ therapy (**Figure 1F**). Similar results were obtained in the most aggressive GBM patients, who were almost inevitably found to be resistant to TMZ (**Figure 1G**). To extend these observations, we examined CUL4B expression in primary and recurrent gliomas. Both the recurrent gliomas and GBMs showed significantly higher CUL4B expression than primary tumors (**Figures 1H, I**). Moreover, high expression of CUL4B also correlated with poor prognosis in both primary and recurrent glioma patients (**Figures 1J, K**).

Previous studies demonstrate that the recurrent GBM shows a more aggressive behavior due to a phenotypic shift toward the mesenchymal subtype (47). A multitherapy-resistant phenotype is also associated with a mesenchymal profile (48). Using CGGA and GSE72951 cohorts, we found that CUL4B expression was markedly enriched in the mesenchymal subtype (**Figures 1L, M**). Based on the Ivy GAP database, the highest CUL4B expression was observed in microvascular proliferation zone (**Figure 1N**), which was also related to GBM recurrence (49).

MGMT promoter methylation and IDH mutations are strongly associated with TMZ resistance in gliomas (13, 50–52). Therefore, we further analyzed the effect of CUL4B on patient prognosis in different status of MGMT promoter and IDH mutation. We found that patients with high CUL4B showed a poor prognosis regardless of the methylation state of MGMT promoter (**Supplementary Figures 2C, D**). In patients with mutant type or wild IDH, high CUL4B levels always indicated a poor prognosis (**Supplementary Figures 2E, F**). Together, these results suggest that CUL4B plays a critical role in TMZ resistance and may serve as a potential prognostic biomarker.

### CUL4B Drives TMZ Resistance in GBM Cell Lines

**Figure 2:**
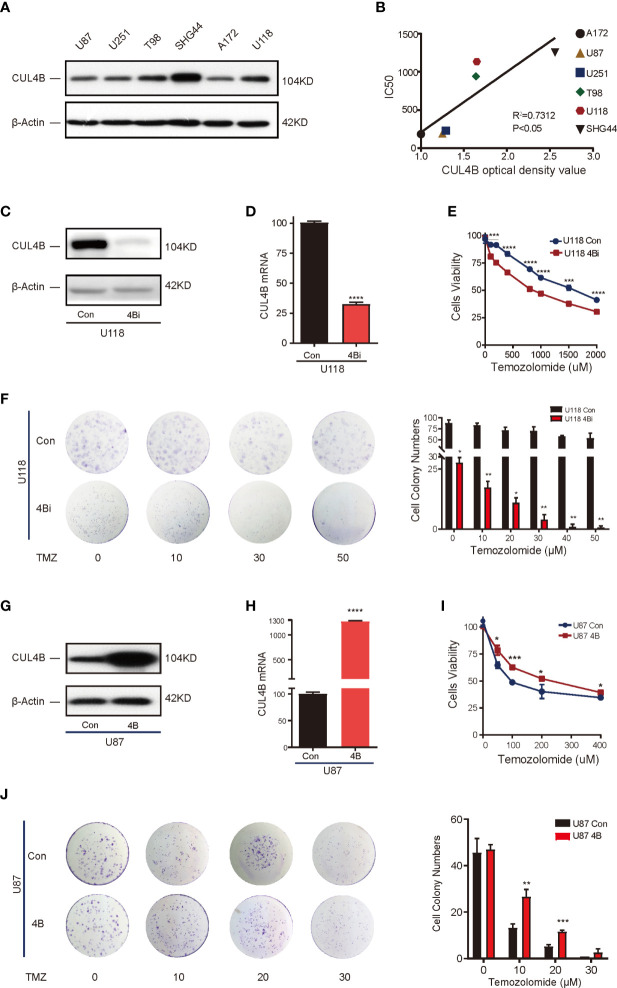
CUL4B drives TMZ resistance in GBM cell lines. **(A)** CUL4B protein levels in indicated cell lines. **(B)** Linear regression analysis of CUL4B expression and IC50 of TMZ in indicated cell lines. **(C, D)** Knockdown efficiency of CUL4B in U118 cells determined by Western blot **(C)** and qRT-PCR **(D)**. **(E)** Cell viability assessed in U118 CUL4B knockdown cells (U118 4Bi) and control (U118 Con) cells. **(F)** Representative images (left panels) and quantification (right panels) of the colony formation assays of U118 Con and 4Bi cells treated with different concentrations of TMZ. **(G, H)** CUL4B expression in indicated U87 cells was determined by Western blot **(G)** and qRT-PCR **(H)**. **(I)** Cell viability assessed in indicated U87 cells. **(J)** Representative images (left panels) and quantification (right panel) of the colony formation assays of indicated U87 cells treated with TMZ at different concentrations. (****P < 0.0001, ***P < 0.001, **P < 0.01, *P < 0.05).

Analysis on patient samples revealed strong association between CUL4B expression and TMZ resistance. To determine the role of CUL4B in TMZ resistance, we first verified if CUL4B level is positively correlated with TMZ resistance in GBM cells. We tested the TMZ sensitivity in 6 human glioma cell lines including A172, U87, U251, T98, U118, and SGH44 cells. As shown in **Supplementary Figure 3**, these cell lines could be divided into two groups according to their IC50 values for TMZ: cell lines with low IC50 values (<300 μM), which included A172 (185.4 μM), U87 (193 μM) and U251 (229.9 μM) cells, and those with high IC50 values (> 300 μM), including T98 (940 μM), U118 (1133 μM) and SHG44 (1253 μM) cells. Western blot analysis showed that cell lines with high IC50 values (T98, U118, and SHG44 cells) had higher CUL4B levels than cell lines with low IC50 values (A172, U87, and U251 cells) (**Figure 2A**). There was a significantly positive correlation between the protein levels of CUL4B and IC50 of TMZ in these cell lines (r^2^ = 0.7312, P<0.05) (**Figure 2B**).

To determine if high CUL4B level is essential for TMZ resistance, we evaluated the effect of knocking down CUL4B on TMZ sensitivity. We chose U118 cells which have high CUL4B and IC50 (high resistance to TMZ) and generated cells with or without CUL4B stably knocked down (designated as U118 4Bi and U118 con, respectively). The efficiency of CUL4B knockdown was verified by Western blot and qRT-PCR (**Figures 2C, D**). CUL4B knockdown U118 cells exhibited a significantly lower IC50 to TMZ than control cells (818.4 μM vs. 1555 μM) (**Figure 2E**). Consistently, colony formation assays confirmed that knockdown of CUL4B impaired survival upon TMZ treatment in U118 cells (**Figure 2F**). These data suggest TMZ resistance requires CUL4B.

To determine whether increased CUL4B expression is sufficient to drive TMZ resistance, we generated U87 cells, which has low CUL4B level and IC50, stably overexpressing CUL4B (designated as U87 4B) and the control cells (designated as U87 con). The expression of CUL4B was determined by Western blot and qRT-PCR (**Figures 2G, H**). CUL4B-overexpressed U87 cells exhibited higher IC50 for TMZ (**Figure 2I**) and stronger colony-forming capacity upon TMZ treatment (**Figure 2J**), indicating CUL4B overexpression promotes TMZ resistance.

### Downregulation of CUL4B in GBM Cells Enhance TMZ Sensitivity *In Vivo*

**Figure 3:**
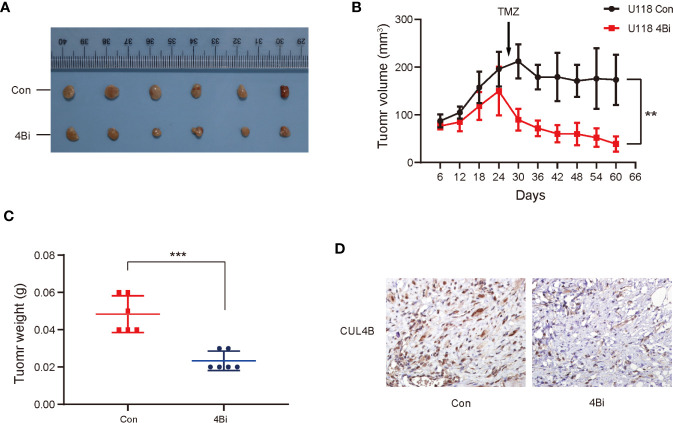
Down-regulation of CUL4B in GBM cells enhance sensitivity of TMZ *in vivo*. Indicated U118 cells were injected into the flanks of nude mice. **(A)** Images of the xenograft tumors derived from U118 Con and U118 4Bi cells. **(B)** Growth curves of tumors from nude mice implanted with indicated cells. **(C)** Tumor weight of the xenografts after dissection. **(D)** Representative images of IHC staining of expression of CUL4B in tumors from nude mice. (***P < 0.001, **P < 0.01).

We further investigated the effect of CUL4B knockdown on TMZ treatment *in vivo*. U118 4Bi cells and control cells were inoculated into BALB/c nude mice. When the tumor size reached approximately 150-200mm^3^, the tumor-bearing mice were treated with TMZ for 5 days per week for two cycles. As expected, knockdown of CUL4B reduced tumor volumes and tumor weight. Importantly, xenografts of U118 4Bi cells displayed higher sensitivity to TMZ and stronger tumor regression than xenograft of control cells (**Figures 3A–C**). Immunohistochemical analysis confirmed that CUL4B expression was significantly lower in tumors derived from U118 4Bi cells than that in tumors derived from control cells (**Figure 3D**). These data demonstrate that CUL4B promotes TMZ resistance *in vivo*.

### CUL4B Suppresses TMZ-Induced Senescence

**Figure 4:**
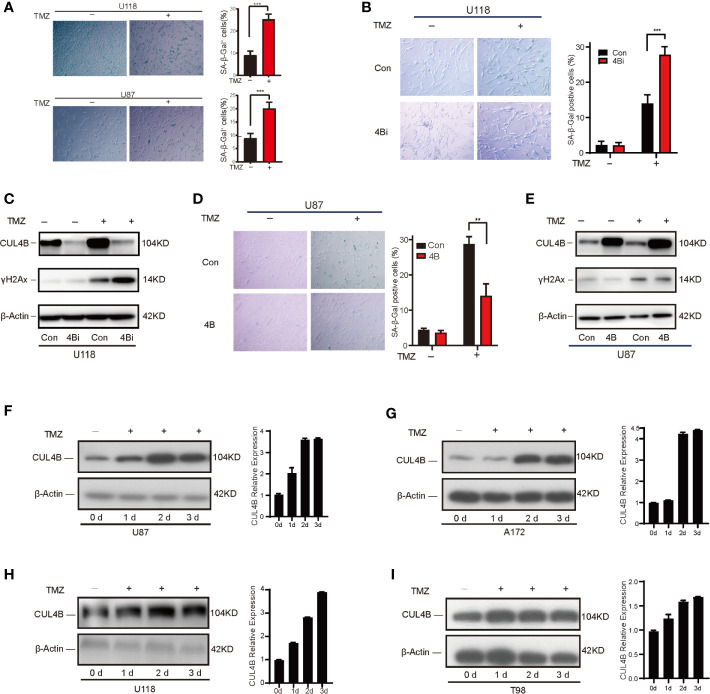
CUL4B suppresses TMZ-induced senescence. **(A)** Senescence induced by TMZ in U118 cells (300μM) and U87 cells (100μM) after 5 days treatment. **(B, C)** SA-β-galactosidase staining **(B)** and γH2Ax levels **(C)** in U118 control and 4Bi cells with and without TMZ (300μM) after 3 days treatment. **(D, E)** SA-β-galactosidase staining **(D)** and γH2Ax level **(E)** in indicated U87 cells treated with and without TMZ (100μM) after 3 days treatment. **(F, G)** TMZ-sensitive U87 **(F)** and A172 **(G)** cells harvested on 0, 1, 2, and 3 days after TMZ addition (100μM). Quantifications of CUL4B expression are shown in the histogram. **(H, I)** TMZ-resistant U118 **(H)** and T98 **(I)** cells harvested on 0, 1, 2, and 3 days after TMZ addition (300μM) and the protein levels of CUL4B were detected. (***P < 0.001, **P < 0.01).

One important mechanism underlying TMZ treatment on glioma is the induction of senescence in TMZ-treated cells. Previously, we found CUL4B could impede stress-induced cellular senescence (42). Thus, we hypothesize that CUL4B promotes TMZ resistance by inhibiting TMZ-induced senescence. To verify this hypothesis, we examined the effect of CUL4B knockdown on senescence. TMZ-treated U118 cells and U87 cells exhibited characteristics of cell senescence, including enlarged and flattened cell bodies, increased biomass, and increased SA-β-gal staining (53) (**Figure 4A**). While knockdown of CUL4B did not affect senescence in untreated cells, TMZ treatment significantly increased the percentage of senescent cells in U118 4Bi cells than that in control cells (**Figure 4B**). Cell senescence is often accompanied by the activation of DNA damage response, and γH2AX is commonly used as a marker of DNA damage (54). As expected, while the basal γH2AX levels were similar in U118 4Bi and control cells, TMZ-induced increase in γH2AX level is significantly stronger in 4Bi cells than that in control cells (**Figure 4C**). Consistently, TMZ-induced senescence and γH2AX upregulation were significantly alleviated in CUL4B overexpressing U87 cells (**Figures 4D, E**). These data together demonstrate that CUL4B suppresses TMZ-induced senescence.

Interestingly, we noted that treatment with TMZ significantly upregulated CUL4B expression (**Figure 4C**), suggesting that TMZ-induced CUL4B upregulation might further promote TMZ resistance. We then examined the CUL4B protein levels following TMZ treatment. As shown in **Figures 4F–I**, the levels of CUL4B protein were remarkably upregulated in all four cell lines (U87, A172, U118 and T98) tested during the 3 days post-incubation period.

### CUL4B Attenuates TMZ-Induced Senescence through Repressing *CDKN1A* Transcription

**Figure 5:**
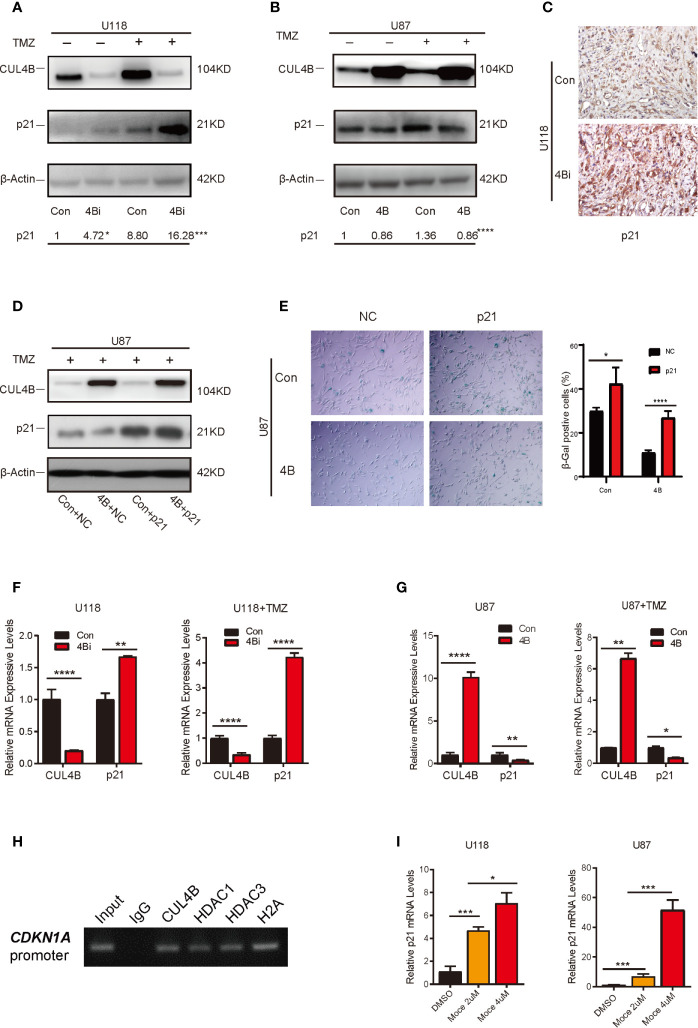
CUL4B attenuates TMZ-induced senescence through repressing *CDKN1A* transcription. **(A, B)** p21 expression in indicated U118 **(A)** and U87 **(B)** cells with or without TMZ treatment. The numbers shown below Western blot images are means of band intensities relative to control without TMZ treatment. Signals on the immunoblots were analyzed by ImageJ, normalized with that of β-actin. **(C)** Representative images of IHC staining of expression of p21 in indicated cells in tumors from nude mice. **(D)** Western blot analysis of indicated proteins in indicated U87cells. **(E)** SA-β-galactosidase staining assays of indicated U87 cells after TMZ treatment. **(F, G)** qRT-qPCR analysis of p21 mRNA expression in U118 cells **(F)** and U87 cells **(G)** with or without TMZ treatment. **(H)** ChIP assays of the recruitment of indicated proteins at the p21 promoter in U87 cells. **(I)** Effects of histone deacetylase (HDAC) inhibitors Mocetinostat (Moce) on p21 mRNA levels in U118 and U87 cells. (****P < 0.0001, ***P < 0.001, **P < 0.01; *P < 0.05).

Wqe next investigated the mechanism through which CUL4B regulate TMZ-induced senescence in GBM cells. Previous studies showed that p21, a cyclin-dependent kinase inhibitor encoded by *CDKN1A*, plays a vital role in the senescence of glioma cells (55–58). We previously found that CUL4B complex interacts and coordinates with the SIN3A-HDAC complex to repress *CDKN1A* transcription in HEK293 and HeLa cells (46). We, therefore, examined the effect of CUL4B on p21 expression in GBM cells. TMZ treatment increased p21 protein levels in both U118 and U87 cells (**Figures 5A, B**). Importantly, both the basal and TMZ-induced p21 expression were significantly enhanced in U118 4Bi cells (**Figure 5A**). TMZ-treated CUL4B knockdown xenografts also exhibited significantly elevated p21 level compared with TMZ-treated control xenografts (**Figure 5C**). Though overexpression of CUL4B in U87 cells did not affect the basal levels of p21, it markedly inhibited the upregulation of p21 induced by TMZ (**Figure 5B**). To determine the role of p21 in CUL4B-regulated cell senescence upon TMZ treatment, we overexpressed p21 in U87 4B cells (**Figure 5D**). SA-β-gal staining showed that p21 overexpression could restore the TMZ-induced senescence in U87 4B cells (**Figure 5E**). Altogether, these results indicate that CUL4B attenuates TMZ-induced senescence at least partially by downregulating p21 expression.

We then determined the mechanisms through which CUL4B inhibits p21 expression. Both the basal and TMZ-induced p21 mRNA levels were increased in CUL4B knockdown GBM cells, and reduced in CUL4B overexpressed GBM cells (**Figures 5F, G**), suggesting that CUL4B represses p21 expression at transcription level. The ChIP assays showed that CUL4B, HDAC1 and HDAC3 could directly bind to the promoter of *CDKN1A* gene in U87 cells (**Figure 5H**). Moreover, treatment with HDACs inhibitor Mocetinostat significantly increased p21 mRNA levels in a dose-dependent manner in both U87 and U118 cells (**Figure 5I**). These data indicate that CUL4B coordinate HDAC1/3 to co-occupy the *CDKN1A* promoter and repress p21 transcription in GBM cells.

## Discussion

The chemotherapy of high-grade gliomas rests on treatment with the anticancer drug TMZ, which is the most widely and effective chemotherapy drug in adults (17). However, due to the inherent or induced resistance to TMZ therapy, recurrence is inevitable and often poses major challenges for clinical GBM management. Thus, more comprehensive understanding of the mechanisms of TMZ resistance and novel therapeutic targets are urgently needed for the clinical management of GBM. CUL4B has been shown to participate in several cancer-associated processes including chemoresistance. Disturbing CUL4B expression could increase sensitivity to chemotherapy in lymphoblastoid cells, non-small-cell lung cancer cells, osteosarcoma and bladder cancer cells (29, 39, 40). Though CUL4B was reported to be involved in the proliferation and migration of GBM cells (41), the role and mechanism of CUL4B in TMZ resistance have rarely been studied.

In this study, our *in vitro* and *in vivo* experiments, complemented by analysis of clinical datasets from CGGA and GEO public database, demonstrated that CUL4B might be a novel key molecule involved in TMZ resistance in GBM cells. We found that CUL4B level was upregulated in glioma tissues and a higher CUL4B level was associated with poor overall survival in glioma patients. In particular, the CUL4B expression was significantly increased in recurrent GBM patients who are insensitive to TMZ treatment than that in primary GBMs. Moreover, we revealed that high CUL4B expression correlated with poor survival in GBM patients receiving TMZ therapy, suggesting that GBM patients with low level of CUL4B would benefit from TMZ based therapy. Consistent with the above findings, ectopic expression of CUL4B in TMZ sensitive GBM cells promoted resistance to TMZ, whereas knockdown of CUL4B sensitized TMZ refractory cells to TMZ. Altogether, our results identify CUL4B can be considered as a potential biomarker for predicting prognosis in GBMs and may be a potential therapeutic target for GBM patients receiving TMZ therapy.

Forcing the cancer cells to undergo apoptosis is an import mechanism by which current cancer therapies, including chemotherapy, irradiation, immunotherapy, exert their antitumor effect (59). However, cancer cells can avoid this scenario and survive with anticancer therapy due to naturally apoptosis resistance. Therefore, exploring the strategy of cancer therapy other than inducing apoptosis in these cancer cells is critically important. Cell senescence is generally regarded as a tumor suppressive event, both by preventing cancer cell proliferation and suppressing malignant progression (60). Chemotherapy is a well-known inducer of senescence. Moderate chemotherapy doses are more likely to cause senescence and higher doses more likely to cause cell death (60). It has been shown most genotoxic drugs used at clinical concentrations trigger cellular senescence of cancer as well as of tumor microenvironment cells  (61). In addition to inhibiting cell proliferation, therapy induced senescence (TIS) and senescence-associated secretory phenotype (SASP) also promote anti-tumor immune response (62). Thus, TIS is one of the key determinants of tumor response to therapy  (61, 63). Indeed, numerous studies show that expression of senescence-associated growth regulatory genes in tumor cells has significant prognostic implications (63). Of note, induction of senescence is often intended in cancer therapy, as lower doses than for the induction of cell death are required and severe side effects including immunosuppression, fatigue, anemia, nausea, diarrhea, and alopecia of therapy are minimized (64). Previous report demonstrated that apoptosis is only a minor pathway, whereas senescence represents a dominant trait triggered by TMZ in GBM cells, indicating enforcing senescence is the key event in improving anticancer action of TMZ in GBMs (7). In this study, we demonstrated that ectopic expression of CUL4B decreased while inhibition of CUL4B enhanced TMZ induced senescence in GBM cells. Together with our previously report that CUL4B attenuated irradiation and oxidative stress-induced senescence in normal human fibroblasts (NHFS) and osteosarcoma cell lines U2OS cells (42), we believe that impeding cellular senescence is an important mechanism through which CUL4B promotes TMZ resistance in GBMs.

p21, encoded by *CDKN1A* gene, was first identified as a CDK regulator that suppresses cell cycle G1/S phase and retinoblastoma protein (RB) phosphorylation (65). For its profound role in halting cellular proliferation, p21 usually acts as a tumor suppressor and is implicated in response to many cancer treatments (65, 66). Therefore, inducing p21 expression could be an effective way to prevent tumor growth and metastasis (65). However, recent studies revealed that under certain conditions, p21 could promote cellular proliferation and oncogenicity, suggests that it can act as a tumor suppressor or as an oncogene (65, 66). p21 functions importantly in senescence and is considered as a typical senescence marker (54). Senescent cells express high levels of p21 (67), and knockout or overexpression of p21 is sufficient to bypass or induce senescence (68, 69). Previous studies also establish an active role of p21 in promoting therapy-induced senescence, indicating p21 as a key molecular mediator of therapy-induced senescence (63, 66, 69, 70). TMZ-induced senescence is also dependent on sustained p21 induction (7, 71, 72). Our results showed that TMZ induced p21 expression is negatively regulated by CUL4B in GBM cell lines and xenograft tumors. The decreased cellular senescence in TMZ-treated CUL4B-overexpressed cells is partly mediated by p21, since expression of exogenous p21 partly restored the level of senescence induced by TMZ. These results indicate that CUL4B promotes TMZ resistance at least partly by negatively regulating p21 induced senescence.

Since p21 has a crucial role in cell cycle arrest, expression level of p21 should be tightly controlled. p53 is a major regulator of p21 transcription. Apart from p53, a variety of other factors including KLF4/6, CDX2, Sp1/Sp3, Smads, Ap2, signal transducers and activators of transcription (STAT), BRCA1, E2F-1/E2F-3, and CAAT/enhancer binding protein α and β also known to be involved in p21 transcription (66, 73). Epigenetic silencing, such as DNA methylation, histone H3 methylation, and histone deacetylation, is another important mode of negative regulation of p21 transcription (73, 74). So strategies to re-expressing epigenetically silenced p21 may induce cell senescence or apoptosis and could be used for cancer treatment. Several anticancer agents such as histone deacetylase (HDAC) inhibitors function, at least partly, through their ability to promote the induction of p21 (75). Our previous study showed that CUL4B negatively regulated p21 transcription by interacting with and coordinating SIN3A-HDAC to co-occupy the p21 promoters in HeLa and HEK293 cells (46). In the present study, we also found in GBM cells, both CUL4B and HDAC1/3 bounded to the p21 promoter, CUL4B knockdown and HDAC inhibitor treatment promoted p21 transcription, indicating CUL4B negatively regulates p21 transcription by coordinating HDACs to co-occupy the p21 promoter in GBM cells. Epigenetic alterations are used as biomarkers and play a central role in glioma treatment decisions (76–78). Thus, existing drugs targeting HDAC1/3 may have a potent effect on TMZ therapy for CUL4B overexpressed GBM tumors.

It is important to emphasize that some questions remain unsolved in this study. We found expression of exogenous p21 only partly restores the level of senescence induced by TMZ in CUL4B overexpressed GBM cells, suggesting that in addition to p21 inhibition, other molecular mechanisms are also involved in repressing TMZ induced senescence by CUL4B. Our previous study showed that CUL4B could promote p53 ubiquitination and proteosomal degradation in NHFs exposed to oxidative stress, thus dampening the p53-dependent cellular senescence (42). Another study also revealed that CUL4B interacted and promoted polyubiquitination of p53 for its degradation in HEK293 cells (79). These results indicate p53 is one of target proteins of CUL4B. Given the critical role of p53 in regulation of cellular senescence and cancer treatment (80), it would therefore be worthwhile to define the role of p53 in CUL4B mediated TMZ resistance in GBM cell. This study only focuses the role of CUL4B, alteration of a single candidate biomarker might bring unpredictable effects due the heterogeneity of GBM. Therefore, it is crucial to analyze the relationship between CUL4B and other key regulator of TMZ resistance in future studies. Moreover, CUL4B is frequently overexpressed and functions importantly in cancer cells, an understanding of the mechanism by which CUL4B expression is upregulated such as by TMZ may also be invaluable for CUL4B-targeting cancer therapy.

In summary, the results of this study indicate that CUL4B promotes TMZ resistance in GBM cells by epigenetically repressing *CDKN1A* transcription, CUL4B is a significant clinical prognostic factor and may serve as a promising therapeutic target for the treatment of gliomas. Our study not only reveals a novel mechanism underlying acquired TMZ resistance but also has important implications in the development of treatment strategies for TMZ-resistant GBMs.

## Data Availability Statement

The datasets presented in this study can be found in online repositories. The names of the repository/repositories and accession number(s) can be found in the article/**Supplementary Material**.

## Ethics Statement

The studies involving human participants were reviewed and approved by The Ethical Committee of School of Basic Medical Sciences, Shandong University. The patients/participants provided their written informed consent to participate in this study. The animal study was reviewed and approved by The Ethical Committee of School of Basic Medical Sciences, Shandong University.

## Author Contributions

YZ and YG conceived the study and designed the experiments. XY, XL, LG, FT and YS performed the experiments. MG and HH advised on the study. XY, GS, YZ and YG interpreted the data and prepared the manuscript. YZ and YG supervised the study. All authors contributed to the article and approved the submitted version.

## Funding

This work was supported by National Natural Science Foundation of China (grant nos. 31671427 and 32070712 to YZ and 81902837 to GS) and Youth Interdisciplinary and innovation Research Group of Shandong University (2020QNQT003 to HH).

## Conflict of Interest

The authors declare that the research was conducted in the absence of any commercial or financial relationships that could be construed as a potential conflict of interest.
