# Supplementary material for: CUL4B Promotes Temozolomide Resistance in Gliomas by Epigenetically Repressing CDNK1A Transcription
Source: Front Oncol. 2021 Apr 2;11:638802. doi: 10.3389/fonc.2021.638802 (PMC8050354; doi:10.3389/fonc.2021.638802)
Supplement: Supplementary file 1 [file DataSheet_1.pdf]

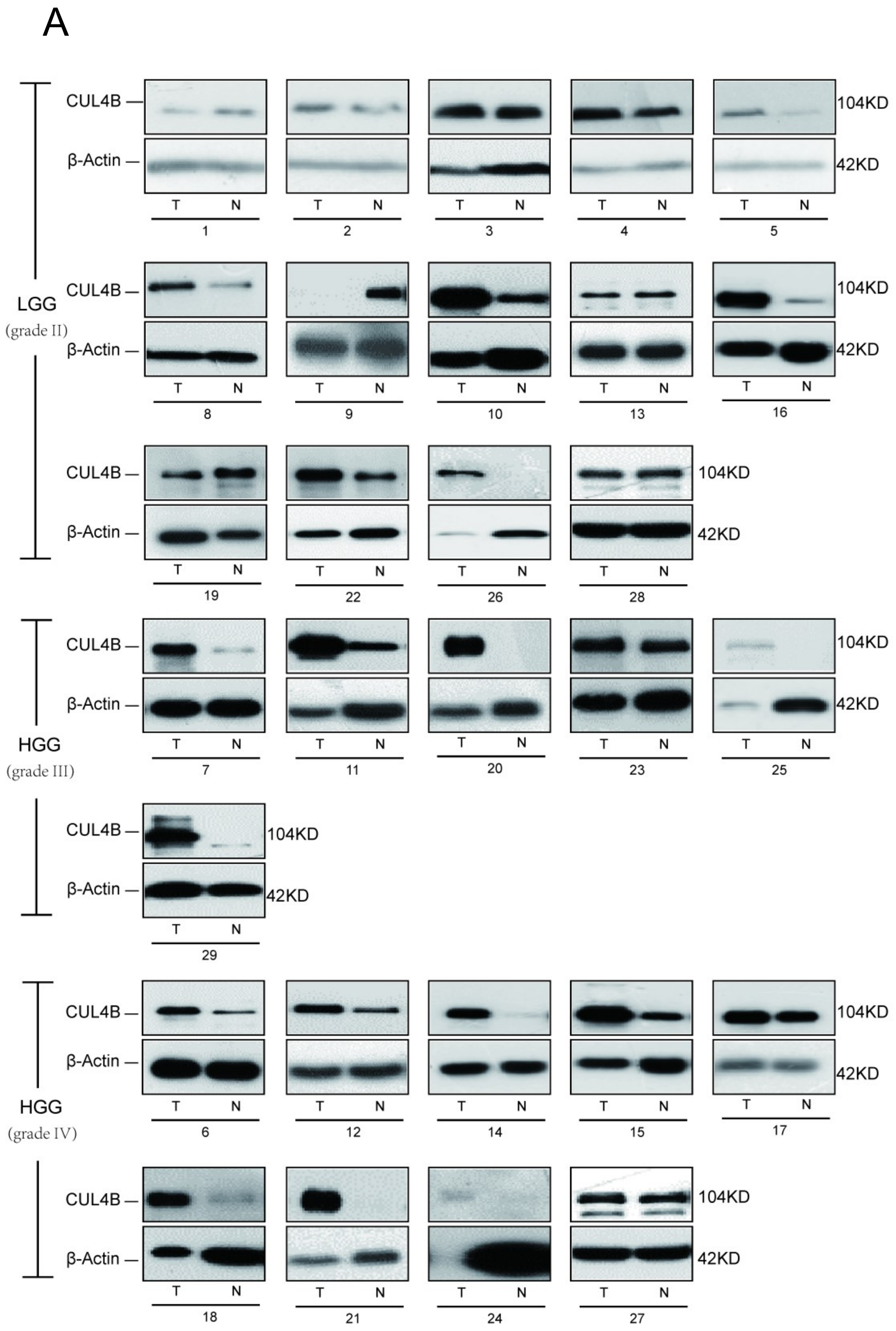

Supplementary figure 1A

Western blot analysis of CUL4B in glioma tissues and paired adjacent non-malignant tissues from 29 patients. (T: tumor tissue; N: paired adjacent non-malignant tissue)

B

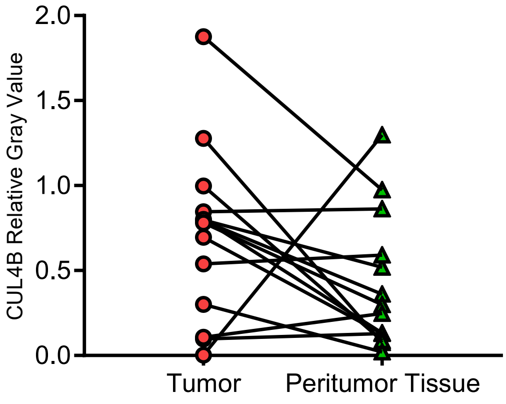

Supplementary figure 1B

Quantitative densitometry ratios of relative CUL4B expression in LGG (grade II) gliomas compared with paired adjacent non-malignant tissues. (LGG: low-grade glioma)
