# Supplementary figures and images for: CUL4B Promotes Temozolomide Resistance in Gliomas by Epigenetically Repressing CDNK1A Transcription
Source: Front Oncol. 2021 Apr 2;11:638802. doi: 10.3389/fonc.2021.638802 (PMC8050354; doi:10.3389/fonc.2021.638802)

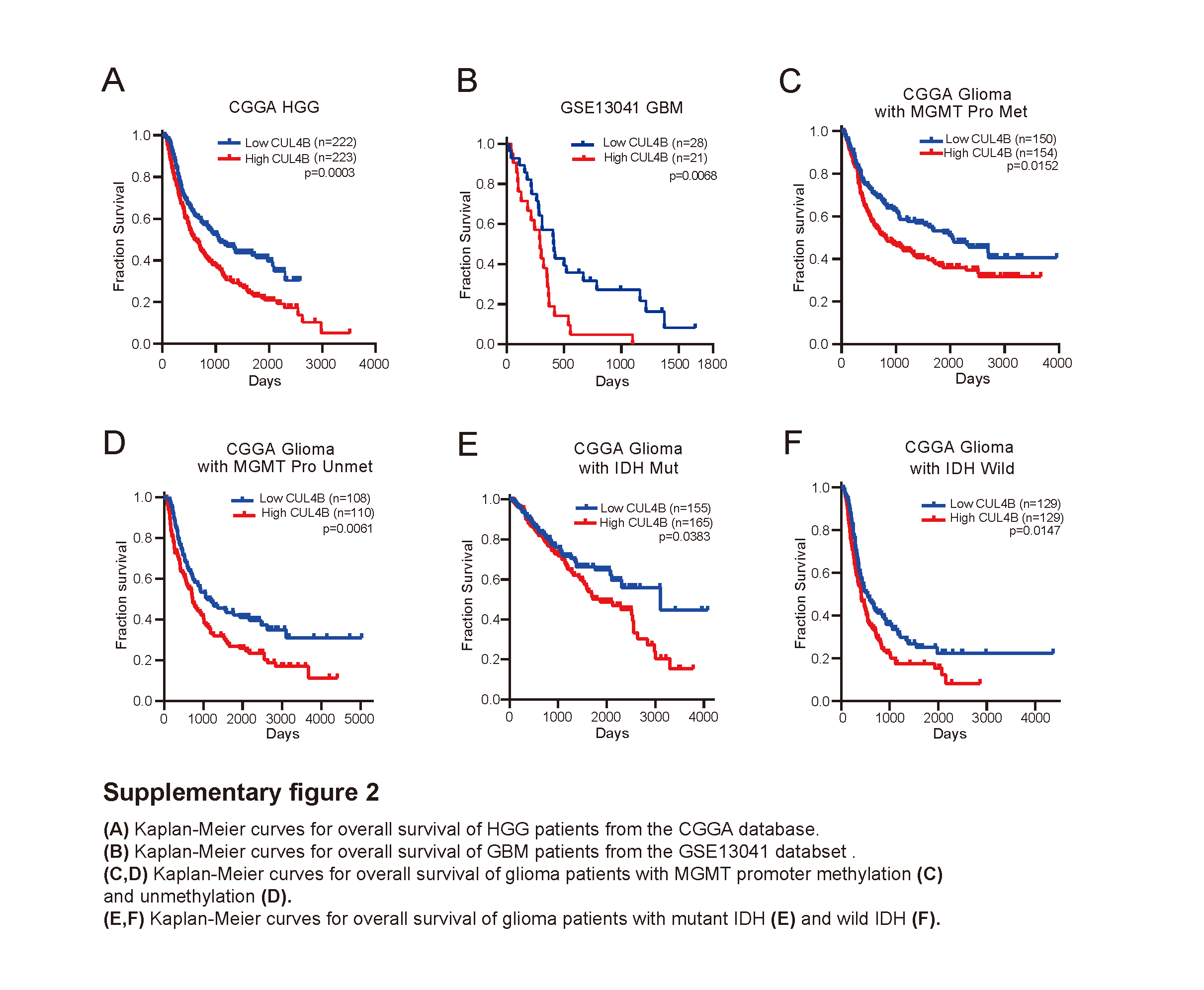

Supplement: Supplementary file 2 [file Image_1.tiff]

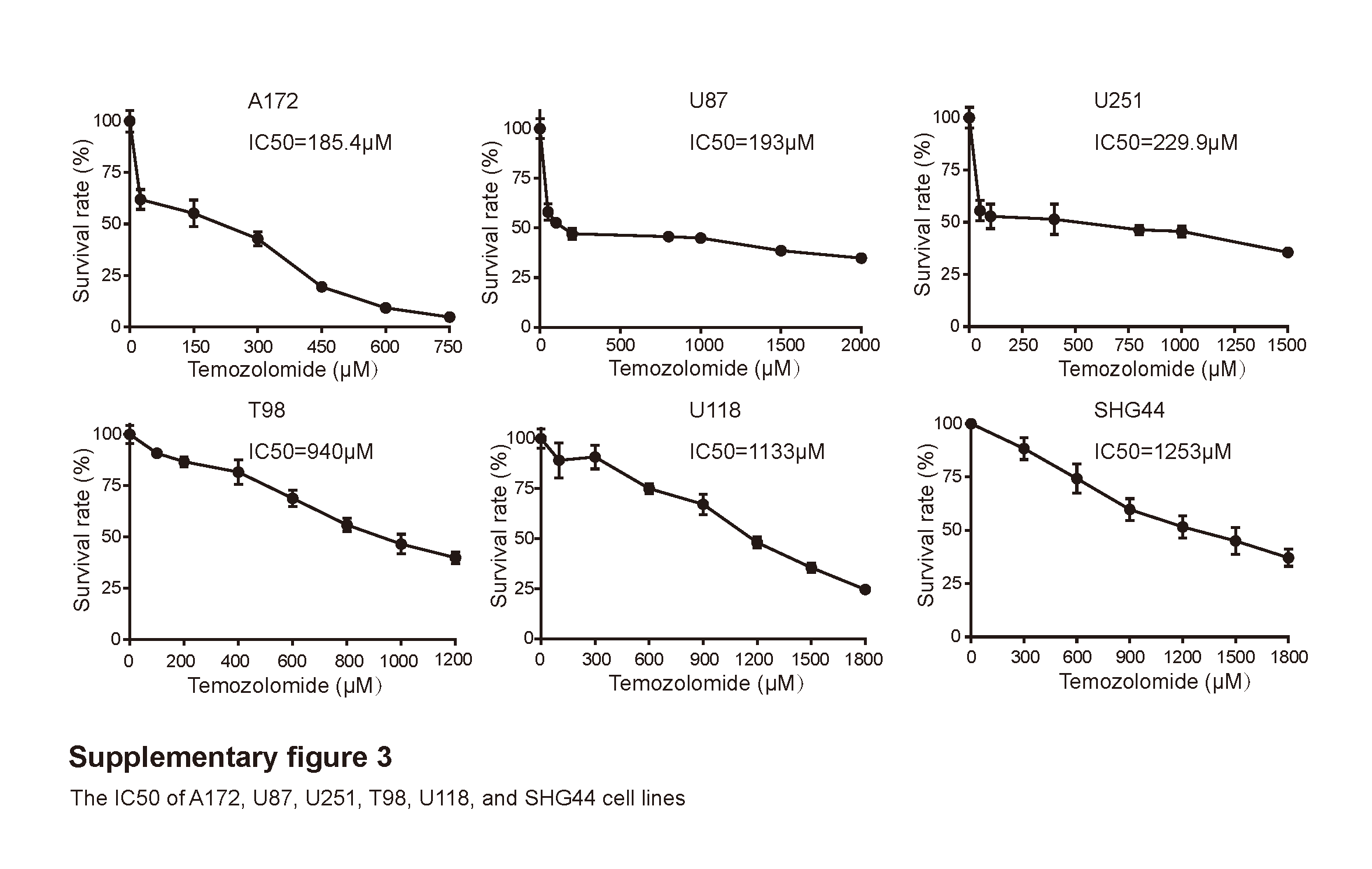

Supplement: Supplementary file 3 [file Image_2.tif]
