# Supplementary material for: CUL4B Promotes Temozolomide Resistance in Gliomas by Epigenetically Repressing CDNK1A Transcription
Source: Front Oncol. 2021 Apr 2;11:638802. doi: 10.3389/fonc.2021.638802 (PMC8050354; doi:10.3389/fonc.2021.638802)
Supplement: Supplementary file 4 [file Table_1.pdf]

Supplementary Table S1 Clinical features of the patients

Patient	Age	Gender	Pathology	Grade
1	31	female	Astrocytoma	II
2	67	female	Astrocytoma	II
3	62	male	Astrocytoma	II
4	44	male	Astrocytoma	II
5	34	female	Oligodendroglioma	II
6	57	male	Glioblastoma	IV
7	36	female	Anaplastic Astrocytoma	III
8	46	male	Oligodendroglioma	II
9	49	female	Astrocytoma	II
10	47	female	Oligoastrocytoma	II
11	46	male	Anaplastic Oligodendroglioma	III
12	46	male	Glioblastoma	IV
13	35	female	Astrocytoma	II
14	46	female	Glioblastoma	IV
15	47	male	Glioblastoma	IV
16	30	female	Oligodendroglioma	II
17	56	female	Glioblastoma	IV
18	67	male	Glioblastoma	IV
19	65	male	Astrocytoma	II
20	39	female	Anaplastic Oligodendroglioma	III
21	58	male	Glioblastoma	IV
22	47	male	Astrocytoma	II
23	57	male	Anaplastic Astrocytoma	III
24	58	male	Glioblastoma	IV
25	52	female	Anaplastic Oligodendroglioma	III
26	46	male	Oligodendroglioma	II
27	50	female	Glioblastoma	IV
28	38	male	Astrocytoma	II
29	59	male	Anaplastic Astrooligodendroglioma	III
